# Supplementary material for: The emergence of cost effective battery storage
Source: Nat Commun. 2019 May 2;10:2038. doi: 10.1038/s41467-019-09988-z (PMC6497676; doi:10.1038/s41467-019-09988-z)
Supplement: Supplementary file 1 — Supplementary Information [file 41467_2019_9988_MOESM1_ESM.pdf]

The Emergence of Cost Effective Battery Storage

Supplementary Information

Comello et al.

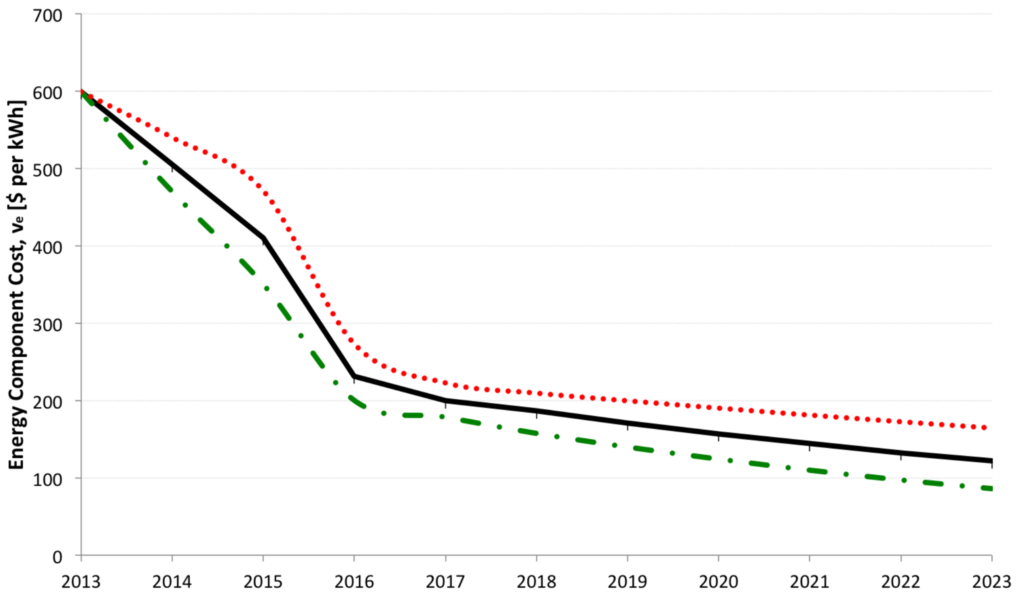

Supplementary Figure 1: *Average, minimum and maximum estimates of unit cost of energy components, v_e, for each year 2013 – 2023. Source data are provided as a Source Data file.*

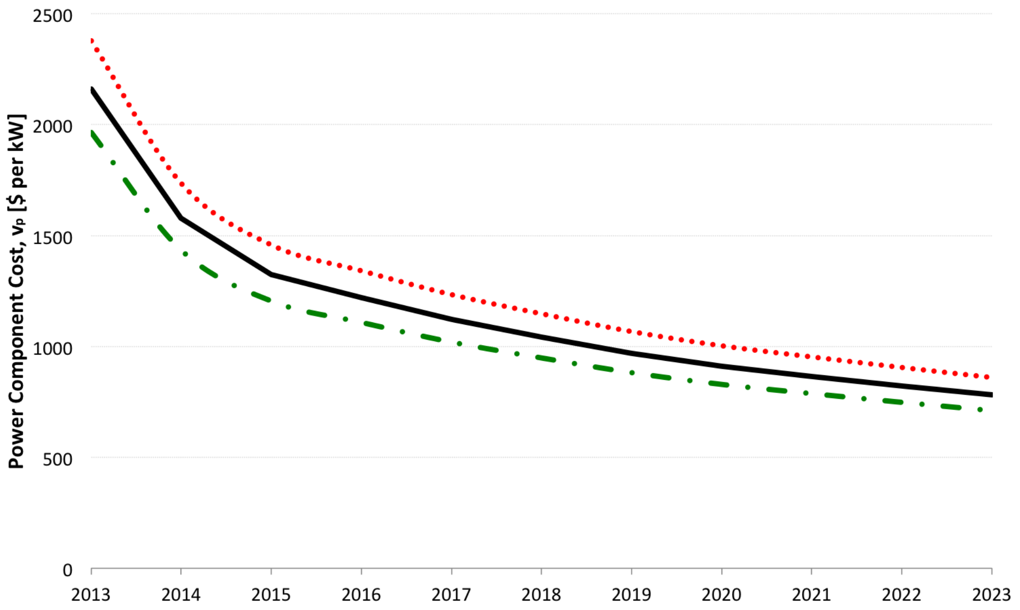

Supplementary Figure 2: *Average, minimum and maximum estimates of unit cost of power components, v_p, for each year 2013 – 2023. Source data are provided as a Source Data file.*

Supplementary Table 1: *Historical, current and projected values of* v_e *in \$ per kWh.*

**Literature Sources**								
Year	(1) GTM Research (2018)	(2) Ardani et al. (2017)	(3) Ardani et al. (2017)	(4) Curry (2017)	(5) Kittner et al. (2017)	(6) de Sisternes et al. (2017	(7) IRENA (2017)	(8) Schmidt et al. (2017)
2013	22%			599				
2014	24%	470		540				
2015	24%		471	350				
2016	14%			273	203	250	200	
2017	14%			209	178	223	188	
2018	10%			191	158	198	176	210
2019	9%			174	140	177	165	200
2020	8%			159	124	157	155	190
2021	8%			145	110	140	145	181
2022	8%			132	97	125	136	173
2023	8%			120	86	111	128	164

Supplementary Table 2: *Using the values provided in Supplementary Table 1, the calculated average, maximum and minimum values of* v_e *per year are presented in \$ per kWh.*

Year	Average	Minimum	Maximum
2013	599	599	599
2014	505	470	540
2015	411	350	471
2016	231	200	273
2017	199	178	223
2018	186	158	210
2019	171	140	200
2020	157	124	190
2021	144	110	181
2022	133	97	173
2023	122	86	164

Supplementary Table 3: *Imputed values of* v_{p} *in \$ per kW, given 2018 baseline of \$1,043 per kW (based on*[17] *project level data) and annual rates of change.*[3]

Year	Reported BOS Annual Cost Declines from GTM Research (2018)	Baseline	Upper Bound Confidence Band +10%	Lower Bound Confidence Band -10%
2013	11%	2161	2377	1964
2014	32%	1577	1735	1434
2015	27%	1325	1458	1205
2016	16%	1219	1341	1108
2017	8%	1122	1234	1020
2018	8%	1043	1147	948
2019	7%	970	1067	882
2020	6%	912	1003	829
2021	5%	866	953	787
2022	5%	823	905	748
2023	5%	782	860	711

Supplementary Table 4: *Input variables and calculated FC, LCOEC, LCOPC for storage installation in Munich, Germany, used to calculate results given in the main text.*

**Input Parameters**			
Parameter	Value	Unit	Ref.
Useful life – power components	30	years	22
Useful life – energy components	10	years	23
Deflator	0.5366		
Energy capacity	k_e	kWh	
Power capacity	k_p	kW	
Unit price for energy component	171	$ per kWh	
Unit price for power component	970	$ per kW	
Unit price for power component, with deflator	520	$ per kW	
Fixed cost	300	$	
Investment tax credit	0%		
Cycles per year	365		13,24,25
System degradation factor	99.0%		22,26,27
Roundtrip efficiency	95.0%		24,28,29
Capital subsidy	0	$ per kWh	
Fixed O&M Cost	0	$ per kW-yr	
Variable O&M Cost	0	$ per kWh	
Fuel Cost	0	$ per kWh	
Cost of Capital	5%		
Federal tax rate	0%		
State tax rate	0%		
Federal Tax Depreciation Method	n/a		
State Tax Depreciation Method	n/a		
**FC, LCOEC and LCOPC Calculations**			
Levelized Fixed Cost (D=4 hours)	0.0158	$ per kWh	
Unit Capacity Cost for Energy Component	0.0672	$ per kWh	
Unit Capacity Cost for Power Component	0.2064	$ per kW	

Supplementary Table 5: *Input variables and calculated FC, LCOEC, LCOPC for storage installation in Los Angeles, California, used to calculate results given in the main text.*

**Input Parameters**			
Parameter	Value	Unit	Ref.
Useful life – power components	30	years	22
Useful life – energy components	10	years	23
Deflator	0.5366		
Energy capacity	k_{e}	kWh	
Power capacity	k_{p}	kW	
Unit price for energy component	171	\$ per kWh	
Unit price for power component	970	\$ per kW	
Unit price for power component, with deflator	520	\$ per kW	
Fixed cost	4000	\$	
Investment tax credit	$\hat{\mathrm{ITC}}(k_{\mathrm{p}}, k_{\mathrm{e}} \mid \bar{G})$		
Cycles per year	365		13,24,25
System degradation factor	99.0%		22,26,27
Roundtrip efficiency	95.0%		24,28,29
Capital subsidy	$\mathrm{SGIP}(k_{\mathrm{p}}, k_{\mathrm{e}})$	\$ per kWh	
Fixed O&M Cost	0	\$ per kW-yr	
Variable O&M Cost	0	\$ per kWh	
Fuel Cost	0	\$ per kWh	
Cost of Capital	5%		
Federal tax rate	0%		
State tax rate	0%		
Federal Tax Depreciation Method	n/a		
State Tax Depreciation Method	n/a		
**FC, LCOEC and LCOPC Calculations**			
Levelized Fixed Cost (D=4 hours)	0.0211	\$ per kWh	
Unit Capacity Cost for Energy Component (D=4 hours)	-0.053	\$ per kWh	
Unit Capacity Cost for Power Component	0.2046	\$ per kW	

Supplementary Note 1: Introduction to the costs of Power Components, Energy Components & Installation

The past, current and projected unit power and energy component costs, v_{p} and v_{e} respectively, are estimated based on a blend of academic and industry publications. Our work uses the so-defined technology scope of electrical energy storage technologies, which clearly defines energy and power components of a (lithium-ion) battery storage system, as defined in prior work.[1] Specifically, this approach separates the battery pack from the ex-works system (inverter, container, battery management system) and the system (transport, installation, commissioning) on a technology and cost basis. These can be found in Table 2 and Figure 1 of the Supplementary Information accompanying Schmidt et al. (2017). Note that their approach to separating battery storage system into its components is consistent with other approaches in the literature.[2–5]

In the present work, the energy components refer to the storage modules, which themselves comprise a series of batteries and cells. The power components refer to the balance of system (BOS), including inverter and related power components, thermal control, hardware, container software subcomponents such as the battery management system. The cost of the energy components is measured on a $ per kWh basis. All power components are measured on a $ per kW basis. Some installation costs, which consist of permitting, inspection/commissioning and workforce mobilization are considered fixed costs, FC, which do not scale with the size of the system. Current installations costs are estimated based on grey literature.

Supplementary Note 2: Unit Cost of Energy Components

Historical, current and projected unit cost of energy component are extracted from multiple sources in the literature.[1,3,6–10] Taken together, these sources provide historical costs of the energy components of a lithium-ion battery system, v_{e}, for each year 2013 to 2017 and offer projected costs from 2018 to 2023. Reported and derived (imputed) values are provided in Supplementary Table 1. The purpose of this table is to display estimates for v_{e} from each

literature source for each year of the years 2013 – 2017 and projections for the years 2018 – 2023. These values are then used to provide minimum, maximum and average estimates of v_e for each year (see Supplementary Table 2). These estimates are then used as the to determine the LCOEC for each year, which is an input to the calculation of LCOES(D) shown in Figure 1 of the main text (the underlying data of which is provided in the Data Source file).

Not every source available to us provided a projection for the cost of v_e for each year to 2023. In such cases, the missing values are imputed using reported or implied annual rates of change. For example, if a literature source provides cost estimates for the years 2016 – 2020 inclusive, then an annual rate of change is calculated based on those years. This rate is then applied to impute each annual unit cost to 2023 for that source. To reiterate, the purpose of imputing annual cost of energy components for 2017 – 2023 is to first create an implied estimate for energy components for each year for the cited source and second, obtain a range of values – minimum, maximum and average – for each projected year to be used as part of the calculation of the range LCOES(D) as shown in Figure 1 of the main text.

Supplementary Table 1 provides a summary of reported v_e prices and imputed annual rates of change of such prices from each literature source. The first source, denoted as (1), provides an annual rate of cost declines for energy components for each year 2013 – 2023 inclusive. This yields an annualized average rate of cost reduction of 9.3% over the entire period[3]. The second resource, denoted as (2) and (3), provides only point estimates for the years 2014 and 2015 respectively (no annual rate of change is determined).

The next literature source estimate, denoted as (4), reports from 2017 ($209 per kWh) and forecasts to 2025 ($100 per kWh).[7] Based on this, an annualized rate of cost reductions is calculated (8.8%) and used to impute the v_e cost from 2018 – 2023 inclusive. The literature source denoted as (5) reports from 2016 ($202 per kWh) and forecasts to 2020 ($124 per kWh).[8] Based on this, an annual (linear) rate of cost reduction is calculated (11.5%) and used to impute the battery pack cost for each year 2018 – 2023 inclusive. Next, source (6) reports from 2016 ($250 per kWh) and offers a point estimate for 2022 ($125 per kWh).[9] Based on this, an annualized rate of cost reductions is calculated (10.9%) and used to impute the battery pack cost for each year 2018 – 2023.

The next source estimate, denoted as (7), dates from 2016 ($250 per kWh) and offers a point estimate for 2030 ($82 per kWh).[10] Based on this, an annual (linear) rate of cost reduction is calculated (6.2%) and used to impute the battery pack cost for each year 2018 – 2023 inclusive. Finally, the literature source (8) forecasts point estimates for 2030 ($117 per kWh) and for 2040 ($72 per kWh).[1] On that basis, an annualized rate of cost reduction is calculated (4.7%) and used to impute the battery pack cost for each year 2018 – 2023 inclusive.

Given the imputed values for v_e, the average, maximum and minimum for each year is taken across all sources. This simple approach provides a range for the cost of the energy components of a battery for each year, as shown in Supplementary Table 2 and Supplementary Figure 1.

The calculations illustrate a rapid decrease in v_e from 2013 – 2016 (annual change of -26.8% for the mean/average case), while maintaining a more conservative forward estimate of 9.6% reductions for the years 2016 – 2023 on a year-over-year basis (mean/average case). In the most aggressive projection for 2016 – 2023, we calculate the annual cost reduction rate of 15.2%. By comparison, the benchmark estimate offered by the Bloomberg New Energy Finance New Energy Outlook 2018 presumes an average 18% annual reduction rate for 2017 to 2030.[11] Further, literature that accounts for the influence of the mechanisms of learning and research and development in the cost reduction of storage, estimate an average of 11.5% from 2016 – 2020, which is also more favorable than the baseline assumption used herewith.[8] In keeping with the concept of learning-by-doing, it has also been demonstrated across multiple technologies, including lithium-ion batteries[1,8] that increased experience with technologies leads to decreased costs and increased production efficiency. Given that global lithium-ion production capacity is planned to expand from 8 GWh per year to over 35 GWh/year in the next 10 years to meet expanding demand, it is arguably a fair presumption that the expanded production base will support ongoing cost decreases.[12,13] Finally, most recent announcements by industry analysts[14] report that the year-end average cost of lithium-ion battery modules for 2018 was $175 per kWh, well inline with the estimates for 2018 – 2019 in our model.

Supplementary Note 3: The Cost of Installation

With respect to installation costs, these costs consisted of those that do not scale with the power or energy capacity of the storage system. This fixed cost, measured in $, jumps from zero to its full amount with system deployment. Contained within this fixed cost are permitting, inspection/commissioning, and workforce mobilization costs.

Academic literature on installation costs is difficult to obtain, thus we rely on grey literature, specifically customer feedback within on-line forums[15] related to Tesla Energy Powerwall actual cost of installations and interviews with three Tesla Energy employees located in Palo Alto, California [personal communication, 2019]. Considering only the fixed costs that would not change depending on the size of the battery or the complexity of the installation, we estimate $400 per system to be appropriate for a residential system.

While this figure may be appropriate for California, it needs adjustment for Germany. Given the differences of labor practices and regulatory environment (e.g. building codes, etc.) between the two jurisdictions a cost adjustment can be made. A benchmark for such an adjustment is found in the literature concerned with cost differentials in residential solar installations.[16] Applying this logic to residential storage installation costs as defined, it is estimated that the fixed cost of installation, the part that is independent of the size of the battery, is $300 (approximately €260) per system in Germany.

Supplementary Note 4: Unit Cost of Power Components

The range of available estimates for the acquisition unit cost power components, υ_{p}, is limited. We therefore rely on project-level data provided by the California Public Utility Commission (CPUC). For the years 2017 – 2018, the CPUC report provides values on a residential project basis that includes (i) the total battery system project cost, (in $), (ii) the project energy capacity (in kWh) and (iii) the project power capacity (in kW). The report is filtered to display only those residential projects that are active (i.e. project across all project phases except for those denoted as suspended or rejected) for the year 2018.[17] This yields 1,579 individual projects. For each yielded project, the unit cost of the power component is determined by calculating:

$$\upsilon_{\mathrm{p}_i} = \frac{(\mathrm{TPC}_i - \mathrm{FC}) - \upsilon_{\mathrm{e}} \cdot k_{\mathrm{e}i}}{k_{\mathrm{p}_i}}.$$

In this equation TPC_i is the total project cost for the i-th project (note that i=1 to 1,579), FC is the installation cost of $400 as estimated above, $k_{\text{e}i}$ is the project energy capacity for the ith project, k_{p_i} is the ith project power capacity and $v_{\text{e}} = \$186$ per kWh as determined in section Supplementary Note 2. Finally, the median value of all v_{p_i} is used to avoid the effects of large values, returning a value for v_{p} of $1,043 per kW. This value is then used as the baseline/benchmark cost estimate for power components for the year 2018.

In order to forecast the changes in cost for the power components of the battery system, we use the published estimates for balance of system (BOS) components by GTM Research, which defines BOS components similarly as we do in this work.[1,3] These BOS values are given in Supplementary Table 3, which are then used to both backcast to 2013 and forecast to 2023, based on the 2018 v_{p} value of 1,043 per kW. Further, error bars of 10% are used for each calculated annual value to create a maximum and minimum value along with the baseline for v_p in each year. Supplementary Figure 2 summarizes the findings.

Supplementary Note 5: Estimates of LCOES: Baseline & Error Bars

Based on the definition of LCOEC, LCOPC, FC and LCOES in the main text and the Methods section, a baseline LCOES function is computed using the average v_e and baseline v_{p} estimates given in Supplementary Table 1 and Supplementary Table 2. For the same figure, lower error bars were determined by the minimum yearly values of v_{e} and v_{p}. Similarly, the maximum yearly values v_{e} and v_{p} were used for upper error bars.

Supplementary Note 6: Comment on Lazard's Levelized Cost of Storage Calculation

The recent publication by Lazard Levelized Cost of Storage Analysis v4.0[13] computes levelized cost of storage values for multiple battery storage technologies and applications. The purpose of the report is to compare the cost of different energy storage technologies under various use-cases. Beyond this main difference and in contrast to our approach – which follows earlier studies[1–3] – Lazard v4.0 views several battery system components as energy rather than power components, for example the battery container, construction cost and the

battery management system. The approach in our study (and those in the antecedent literature cited above) is to view these components scaling proportionally with rated power. The grouping of components is important insofar as the levelized cost of storage corresponding to different duration levels will decrease at a different rate. In addition, Lazard v4.0 fixes the duration of its residential battery systems at 4 hours. Finally, that study arrives at a higher levelized cost of storage due to (i) the inclusion of a corporate tax rate (35%) and (ii) a significantly higher assumed weighted average cost of capital (11.2%).

Supplementary Note 7: Location Specific Application: Germany - Demand, Generation and Other Parameters

German demand profile raw data[18] are used to formulate the demand profile for a representative household Germany residential case. This demand profile assumes a national average residential dwelling and thus is simulation based. Data points are provided in 1-second intervals over a one year time frame, which is converted into monthly and seasonal demand profiles using a simple, purpose-built spreadsheet based tool. The summer season demand profile is based on demand data from March 21 – September 20 inclusive; winter season is September 21 – March 20 inclusive. Representative demand profiles are compressed to 15-minute intervals by taking the 15-minute-specific average across all data provided for that time frame. Results and Table 1 in the main text, along with calculations for optimal battery energy and power components are based on the values provided in the Source Data file (i.e. those tabs with marked as Munich within the title).

The represented German insolation profile uses publicly available data based on satellite observations.[19,20] To determine seasonal generation profiles the national average installed capacity of 6 kW is used for calculations.[21] Summer and winter are defined as before. It is also presumed that installed solar systems are subject to losses including system losses (86%), inverter efficiency (96%) and temperature losses (97%). Results and Table 1 in the main text, along with calculations for optimal battery energy and power components are based on the values provided in the Source Data file (i.e. those tabs with Munich within the title).

A summary of input variables for the Munich, Germany case are provided in Supplementary Table 4. Some parameters in Supplementary Table 4 require additional explanation. In

particular, the parameter representing the Deflator reflects that the assumed useful life of the power components is 30 years, while that of the energy components is only 10 years, though both are assumed to be subject to the same degradation rate of 1% (with a corresponding degradation factor of 99%). The deflator is calculated as:

$$\frac{\sum_{i=1}^{10} x_i \cdot \gamma^i}{\sum_{i=1}^{30} x_i \cdot \gamma^i}. \tag{SI1}$$

This specification reflects that the planning horizon in our model is 10 years, given by the useful life of the energy components, yet the power components still has positive market value at that point in time since it could be combined with two additional cohorts of energy components, each one lasting 10 years. Anticipating the return of the fair market value of the power components that can be obtained at the end of the 10 year period, the effective expenditure for the power components is only v_p scaled by the deflator in expression Equation (SI1).

Finally, the analysis for Germany does not consider income taxes, as there are no tax consequences for a homeowner due to savings related to battery storage investment.
Using $k_\mathrm{e} = 4$ and $k_\mathrm{p} = 1$ as an example, and Equation (1) from the main text, the LCOES(4) amounts to \$0.119 per kWh or 0.105 € per kWh.

Supplementary Note 8: U.S. Public Policy Support for Battery Storage

As defined in Section 48 of the U.S. Internal Revenue Code: 26 CFR §1.48-9, various types of storage facilities are eligible for the ITC as the entire system is then considered renewable energy property when combined with a solar installation. Eligibility is extended to both new solar with storage installations and to storage devices that are retrofitted to existing solar facilities. To be able to claim the full ITC incentive, 100% of the energy used to charge the storage device must come directly from the solar system for each of the first 5 years the storage facility is placed into service. For storage devices charged by other means there will be a proportional reduction on the remaining amount of the ITC claimed. At the same time the IRS requires that in order to be eligible for any ITC related to a storage facility, at least

75% of the electricity that is used to charge the storage facility must come directly from the solar system in any of the first 5 years. Failing to meet this minimum threshold would result in recapture of the entire ITC, effectively removing the incentive altogether.

At a state level, California offers a capital subsidy through its Self Generation Incentive Program (SGIP). In its current form, SGIP compensates storage developers on a $ per Wh basis for the capital costs of storage projects (up to 100%) that are located at the customer site (behind-the-meter or BTM installations). BTM installations are classified as either large-scale storage (> 10 kW) or small small residential storage (< 10 kW and installed at a place of residence). Currently, for small residential storage, the SGIP incentive is $400 per kWh. The incentive decreases in a step-wise function as thresholds of deployment capacity are achieved for both large-scale and small residential classes. Notably, SGIP incentives are reduced as the duration of energy storage (Wh) increases in relation to rated capacity.[33] Importantly, while SGIP cannot be applied to solar facilities, the ITC can be used in combination with SGIP to reduce the cost of storage given that the combined solar and battery storage facilities constitute one system.

Supplementary Note 9: Location Specific Application: California - Demand, Generation and Other Parameters

Raw data for the demand profile in California is provided by the OpenEI dataset.[30,31] Input data is converted into annual representative demand profiles by location. Data is provided in 1-hour intervals over a one year time frame, which is converted into two representative daily demand profiles – summer and winter – using a simple, purpose-built spreadsheet based tool. The summer demand profile is based on data from June – September inclusive; winter is October – May inclusive. Representative demand profiles are expanded to 15-minute intervals through linear interpolation of hourly results. Results and Table 2 in the main text, along with calculations for optimal battery energy and power components are based on the values provided in the Source Data file (i.e. those tabs with the term LA within the title).

For California, raw data for insolation profiles – specifically plane of array irradiance (W per m^2) and cell temperature ($^{\circ}C$) – is used.[32] Installed capacity is determined to be that which provides generation equal in magnitude to the average annual demand. This presumption

is based on the predominance of net-metering regulations that generally restrict installed system capacity to no greater than 100% of annual demand. The resulting installed capacity is 4.85 kW for California (Los Angeles). It is also assumed that installed solar systems are subject to multiple losses, including system losses (86%), inverter efficiency (96%) and temperature losses (97%). Results and Table 2 in the main text, along with calculations for optimal battery energy and power components are based on the values provided in the Source Data file (i.e. those tabs with the term LA within the title). A summary of input variables for the Los Angeles, California case are provided in Supplementary Table 5.
The deflator is as defined in previously within Supplementary Note 7. The applicable ITC and SGIP rules are described in the *Methods* section of main article. Using $k_\mathrm{e} = 4$ and $k_\mathrm{p} = 1$ as an example yields LCOEC of -$0.053 per kWh and a LCOPC of $0.2046 per kW. Combing these results with Equation (1) from main text, the LCOES(4) is $0.006 per kWh.

Supplementary References

[1] Schmidt, O., Hawkes, A., Gambhir, A. & Staffell, I. The future cost of electrical energy storage based on experience rates. *Nature Energy* **2**, – (2017). URL https://www.nature.com/articles/nenergy2017110.

[2] Wu, D., Kintner-Meyer, M., Yang, T. & Balducci, P. Economic analysis and optimal sizing for behind-the-meter battery storage. In *2016 IEEE Power and Energy Society General Meeting (PESGM)*, 1–5 (2016).

[3] GTM Research. U.s. front-of-the-meter energy storage system prices 2018-2022. Tech. Rep., Greentech Media (GTM) Research (2018).

[4] Tervo, E. *et al.* An economic analysis of residential photovoltaic systems with lithium ion battery storage in the united states. *Renewable and Sustainable Energy Reviews* **94**, 1057 – 1066 (2018). URL http://www.sciencedirect.com/science/article/pii/S1364032118304921.

[5] O'Shaughnessy, E., Cutler, D., Ardani, K. & Margolis, R. Solar plus: Optimization of distributed solar pv through battery storage and dispatchable load in residential buildings. *Applied Energy* **213**, 11 – 21 (2018). URL http://www.sciencedirect.com/science/article/pii/S0306261917318421.

[6] Ardani, K. *et al.* Installed cost benchmarks and deployment barriers for residential solar photovoltaics with energy storage: Q1 2016. Tech. Rep., National Renewable Energy Laboratory (2017). NREL/TP-7A40-67474.

[7] Curry, C. Lithium-ion battery costs and market: Squeezed margins seek technology improvements & new business models. Tech. Rep., Bloomberg New Energy Finance (2017).

[8] Kittner, N., Lill, F. & Kammen, D. Energy storage deployment and innovation for the clean energy transition. *Nature Energy* **2**, – (2017). URL http://dx.doi.org/10.1038/nenergy.2017.125.

[9] de Sisternes, F. J., Jenkins, J. D. & Botterud, A. The value of energy storage in decarbonizing the electricity sector. *Applied Energy* **175**, 368 – 379 (2016). URL http://www.sciencedirect.com/science/article/pii/S0306261916305967.

[10] IRENA. Electricity storage and renewables: Cost and markets to 2030. Tech. Rep., International Renewable Energy Agency (2017). ISBN 978-92-9260-038-9.

[11] BNEF. New energy outlook 2018. Tech. Rep., Bloomberg New Energy Finance (2018).

[12] IEA. Global ev outlook 2018. Tech. Rep., OECD/International Energy Agency (2018).

[13] Lazard. Lazard's levelized cost of storage analysis. Tech. Rep., Lazard Ltd. (2018). Version 4.0.

[14] Bloomberg LP. Bnef brief: Lithium battery prices fall 18 percent (2018). URL `https://www.bloomberg.com/news/videos/2018-12-21/bnef-brief-lithium-battery-prices-f`[illegible]

[15] Tesla Forums. Total cost of install of powerwall 2 (2019). URL `https://forums.tesla.com/forum/forums/total-cost-install-powerwall-2`.

[16] Seel, J., Barbose, G. & Wiser, R. An analysis of residential pv system price differences between the united states and germany. Tech. Rep., Lawrence Berkeley National Laboratory (2014). LBNL-6614E.

[17] CPUC. California self generation incentive program (sgip) weekly statewide report @ONLINE (2018). URL `https://www.selfgenca.com/home/resources/`. Last accessed 29 April 2018.

[18] Tjaden, T., Bergner, J., Weniger, J. & Quaschning, V. Representative electrical load profiles of residential buildings in germany with a temporal resolution of one second. Tech. Rep. (2015).

[19] Pfenninger, S. & Staffell, I. Long-term patterns of european pv output using 30 years of validated hourly reanalysis and satellite data. *Energy* **114**, 1251 – 1265 (2016). URL `http://www.sciencedirect.com/science/article/pii/S0360544216311744`.

[20] Pfenninger, S. & Staffell, I. Renewables.ninja. https://www.renewables.ninja/ (2018).

[21] IRENA. Irena cost and competitiveness indicators: Rooftop solar pv. Tech. Rep., International Renewable Energy Agency (2017). ISBN 978-92-9260-037-2.

[22] Fisher, M. J. & Apt, J. Emissions and economics of behind-the-meter electricity storage. *Environmental Science & Technology* **51**, 1094–1101 (2017).

URL http://dx.doi.org/10.1021/acs.est.6b03536. PMID: 28001057, http://dx.doi.org/10.1021/acs.est.6b03536.

[23] Dufo-Lopez, R. Optimisation of size and control of grid-connected storage under real time electricity pricing conditions. *Applied Energy* **140**, 395 – 408 (2015). URL //www.sciencedirect.com/science/article/pii/S0306261914012616.

[24] Julch, V. Comparison of electricity storage options using levelized cost of storage (lcos) method. *Applied Energy* **183**, 1594 – 1606 (2016). URL http://www.sciencedirect.com/science/article/pii/S0306261916312740.

[25] Wu, D., Kintner-Meyer, M., Yang, T. & Balducci, P. Analytical sizing methods for behind-the-meter battery storage. *Journal of Energy Storage* **12**, 297 – 304 (2017). URL http://www.sciencedirect.com/science/article/pii/S2352152X17300129.

[26] Peterson, S. B., Apt, J. & Whitacre, J. Lithium-ion battery cell degradation resulting from realistic vehicle and vehicle-to-grid utilization. *Journal of Power Sources* **195**, 2385 – 2392 (2010). URL http://www.sciencedirect.com/science/article/pii/S0378775309017443.

[27] Xu, B., Oudalov, A., Ulbig, A., Andersson, G. & Kirschen, D. S. Modeling of lithium-ion battery degradation for cell life assessment. *IEEE Transactions on Smart Grid* **9**, 1131–1140 (2018).

[28] DOE/EPRI. Doe/epri electricity storage handbook in collaboration with nreca. Tech. Rep., Sandia National Laboratory (2015). SAND2015-1002.

[29] CARMEN. C.a.r.m.e.n. e.v. marktubersicht batteriespeicher: Ein informationsangebot. Tech. Rep., C.A.R.M.E.N (2018).

[30] NREL. Residential base demand profile based on tmy3 (2016). URL http://en.openei.org/datasets/dataset/commercial-and-residential-hourly-load-profile

[31] NREL. Building america house simulation protocols. Tech. Rep., National Renewable Energy Laboratory (2010). NREL/TP-550-49426.

[32] NREL. Pvwatts version 5.2.0. http://pvwatts.nrel.gov/index.php (2015).

[33] CPUC. 2017 self-generation incentive program handbook v3. Tech. Rep., California Public Utilities Commission (2017).
